# Genome-Wide Analysis of Histone H3 Lysine9 Modifications in Human Mesenchymal Stem Cell Osteogenic Differentiation

**DOI:** 10.1371/journal.pone.0006792

**Published:** 2009-08-27

**Authors:** Jiang Tan, Jun Lu, Wei Huang, Zhixiong Dong, Chenfei Kong, Lin Li, Lina Gao, Jianhua Guo, Baiqu Huang

**Affiliations:** 1 The Key Laboratory of Molecular Epigenetics of Ministry of Education, Institute of Genetics and Cytology, Northeast Normal University, Changchun, People's Republic of China; 2 The Key Laboratory for Applied Statistics of Ministry of Education, Institute of Mathematic and Statistic, Northeast Normal University, Changchun, People's Republic of China; McMaster University, Canada

## Abstract

Mesenchymal stem cells (MSCs) possess self-renewal and multi-lineage differentiation potentials. It has been established that epigenetic mechanisms such as histone modifications could be critical for determining the fate of stem cells. In this study, full human genome promoter microarrays and expression microarrays were used to explore the roles of histone modifications (H3K9Ac and H3K9Me2) upon the induction of MSC osteogenic differentiation. Our results revealed that the enrichment of H3K9Ac was decreased globally at the gene promoters, whereas the number of promoters enriched with H3K9Me2 was increased evidently upon osteogenic induction. By a combined analysis of data from both ChIP-on-chip and expression microarrays, a number of differentially expressed genes regulated by H3K9Ac and/or H3K9Me2 were identified, implicating their roles in several biological events, such as cell cycle withdraw and cytoskeleton reconstruction that were essential to differentiation process. In addition, our results showed that the vitamin D receptor played a trans-repression role via alternations of H3K9Ac and H3K9Me2 upon MSC osteogenic differentiation. Data from this study suggested that gene activation and silencing controlled by changes of H3K9Ac and H3K9Me2, respectively, were crucial to MSC osteogenic differentiation.

## Introduction

Embryonic stem (ES) cells are pluripotent cells derived from blastocysts that can differentiate into almost all cell lineages in vivo [1]–[3]. Since the use of ES cells in research as well as in therapeutics is encumbered by ethical considerations, a great deal of attention has been turned to the derivation of stem cells from non-embryonic origins, which provide investigators with an invaluable cell source to study cell and organ development [4], [5]. Such stem cells have been identified in many organ tissues, including hematopoietic, neural, gastrointestinal, epidermal, hepatic and mesenchymal stem cells (MSCs) [6], [7]. MSCs show properties shared by embryonic stem cells; they have the self-renewal potential and can differentiate into several cell lineages including osteoblasts, chondrocytes, adipocytes and myoblasts [8]. MSCs have demonstrated efficacy in multiple types of cellular therapeutic strategies, including applications in treating children with osteogenesis imperfecta, hematopoietic recovery, and bone tissue regeneration strategies [4], [9]. In contrast to diverse and growing information concerning MSCs and their uses in cell-based strategies, the mechanisms governing MSC self-renewal and multi-lineage differentiation are not well understood and remain an active area of investigation [10], [11].

Modification of nucleosomal core histones is thought to be a prevalent epigenetic mechanism in eukaryotic gene regulation, most likely through modulation of chromatin structure [12]. More than two dozens of site-specific histone modifications have been described [13], [14], and the acetylation and methylation of lysine residues in the tails of nucleosomal histones have been shown to exert crucial influences on chromatin packaging and gene expression [15], [16]. In general, acetylation of Lysine9 of histone H3 (H3K9) correlates with gene activation, whereas enrichment of H3K9 methylation is associated with gene silencing [16]. There are three distinctive methylation states of histone H3K9, i.e., mono-, di-, and tri-methylation [17]. The pericentric heterochromatin is enriched for trimethylated H3K9 (H3K9Me3), while centromeric regions are enriched for the dimethylated H3K9 (H3K9Me2) [18], [19]. The establishment of specific gene expression patterns during stem cell differentiation is a result of subtly elaborated control of activation/silencing of large numbers of genes [20]–[23]. To date, the profiles of histone modifications during stem cell differentiation process, which may be tightly associated with the gene expression patterns, have not been extensively studied.

DNA microarray technology has made it possible to profile and quantify the expression of thousands of genes simultaneously [24]. Although the major use of DNA microarrays has been for mRNA expression profiling, there are other applications [25]. Recently, a technical method for genomic mapping of histone modifications in vivo has been developed, allowing researchers to obtain a broader view of the distributions of histone modifications. This method, known as “ChIP-on-chip”, is based on chromatin immunoprecipitation (ChIP) assay, and identifies the enriched DNA fragments by hybridizing to microarrays with probes corresponding to genomic regions of interest [26]–[28].

The aim of this study was to investigate the effects of genomic changes in H3K9Ac and H3K9Me2 modifications at gene promoter regions upon human MSC osteogenic differentiation, by employing strategies based on ChIP-on-chip and expression microarray methods. By linking data from both ChIP-on-chip and expression microarrays, a series of differentially expressed genes regulated by changes of H3K9Ac and H3K9Me2 were identified. Furthermore, we found these genes participated in several cellular pathways and biological events that were essential to MSC osteogenic differentiation. Overall, results from this study represent the first global view of the functional relationships between modifications of H3K9 and gene expression in human MSC differentiation, and suggested that gene activation and silencing controlled by changes of H3K9Ac and H3K9Me2, respectively, may be crucial to MSC osteogenic differentiation.

## Results

### Induction of human MSC osteogenic differentiation

**Figure 1:**
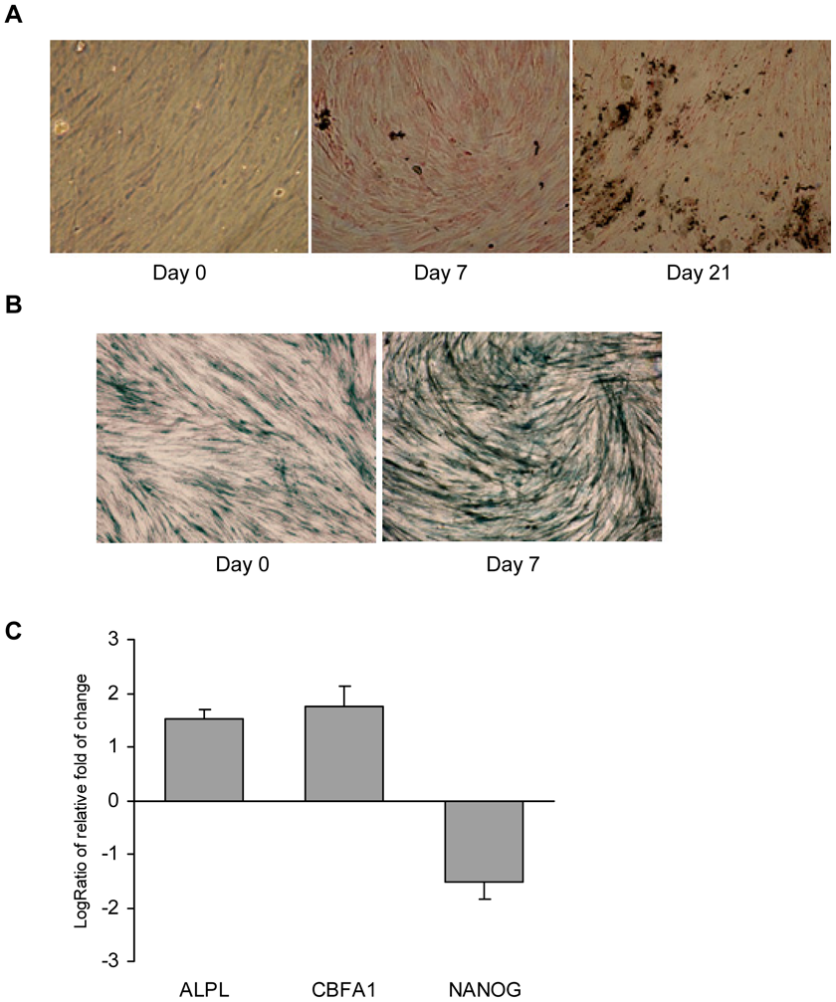
Identification of human MSC osteogenic differentiation. (A) Detection of calcium deposition by von kossa staining from 0 day to 21 days. (B) Determination of early osteogenic marker alkaline phosphatase (ALP) activity by staining in undifferentiated MSCs (*left*), and MSCs 7 days after osteogenic induction (*right*). (C) Detection of expression profiles of MSC osteogenesis marker genes at day 7 of osteogenic induction. Osteogenic specific genes, ALPL and CBFA1, were up-regulated, whereas the stemness marker gene NANOG was down-regulated 7 days after induction of osteogenic differentiation. Standard error bars of three individual experiments are indicated.

Mesenchymal stem cells (MSCs) were derived from human bone marrow, and the MSCs of the fifth generation were induced to osteogenic differentiation by adding osteogenic differentiation medium, as previously described [10]. Under this condition, calcium deposition was detected by von kossa staining at day 7 after osteogenic induction, and the calcium deposition level was increased markedly after 21 days (Figure 1A). Moreover, a significantly increased level of the alkaline phosphatase enzyme (ALPL) was observed at day 7 of osteogenic induction, compared with the control cultures (Figure 1B). Real-time RT-PCR analyses showed that the expression of marker genes of MSC osteogenic differentiation, i.e., ALPL and CBFA1, increased significantly, whereas the marker gene NANOG representative of the stemness of the cells was down-regulated at day 7 of osteogenic differentiation induction (Figure 1C). Thus, day 7 of the induction was determined the transit point of osteogenic differentiation onset of MSCs under this experimental condition, and this time point had been used throughout the study.

### Generation of histone modification profiles at gene promoter regions by using ChIP-on-chip

**Figure 2:**
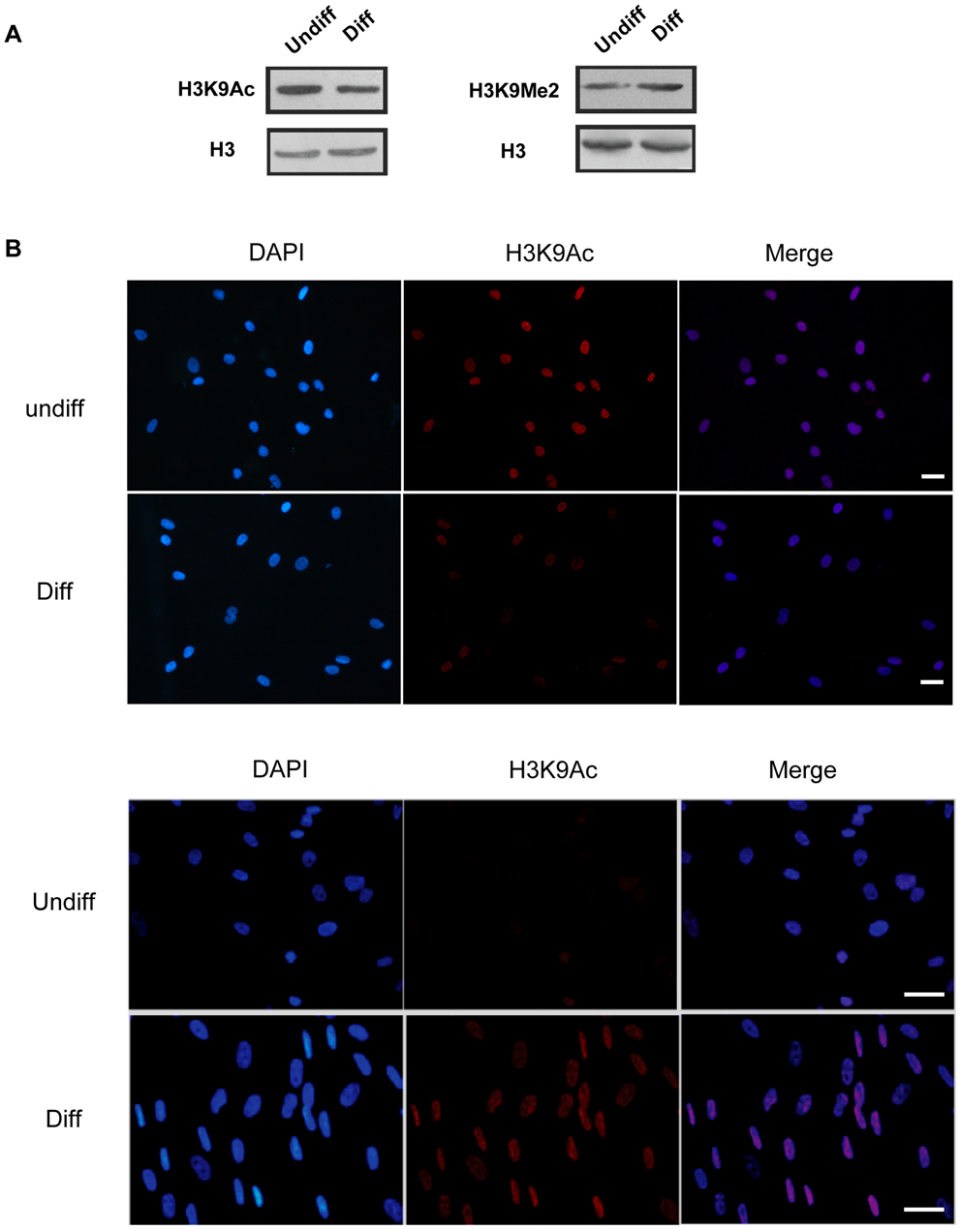
Dynamic modifications of H3K9 in MSC osteogenic differentiation. (A) Western blots comparing global acetylation and dimethylation of H3K9 in undifferentiated and osteogenic differentiating MSCs. (B) Immunofluorescence analysis of H3K9Ac/H3K9Me2 in undifferentiated and osteogenic differentiating MSCs. DNA was stained by DAPI (blue); H3K9Ac and H3K9me2 were detected by the second antibody with Alexa 546 (red). The results indicated that the H3K9Ac level was decreased, whereas the H3K9Me2 level was increased remarkably 7 days after osteogenic differentiation. Scale bar represents 30 μm.

It has been known that the mammalian epigenome undergoes global remodeling during early development [29], and high acetylation of H3K9 correlates with euchromatin, whereas the enrichment of H3K9Me2 is associated with heterochromatin in centromeric regions [18], [19]. We were then interested in addressing whether changes of H3K9Ac and H3K9Me2 occurred during MSC osteogenic differentiation. Global levels of H3K9Ac and H3K9Me2 were assessed by western blotting and immunofluorescence, and we found that the level of H3K9Ac was decreased globally (Figure 2A and B), while the H3K9Me2 level was increased 7 days after osteogenic differentiation (Figure 2A and B).

**Figure 3:**
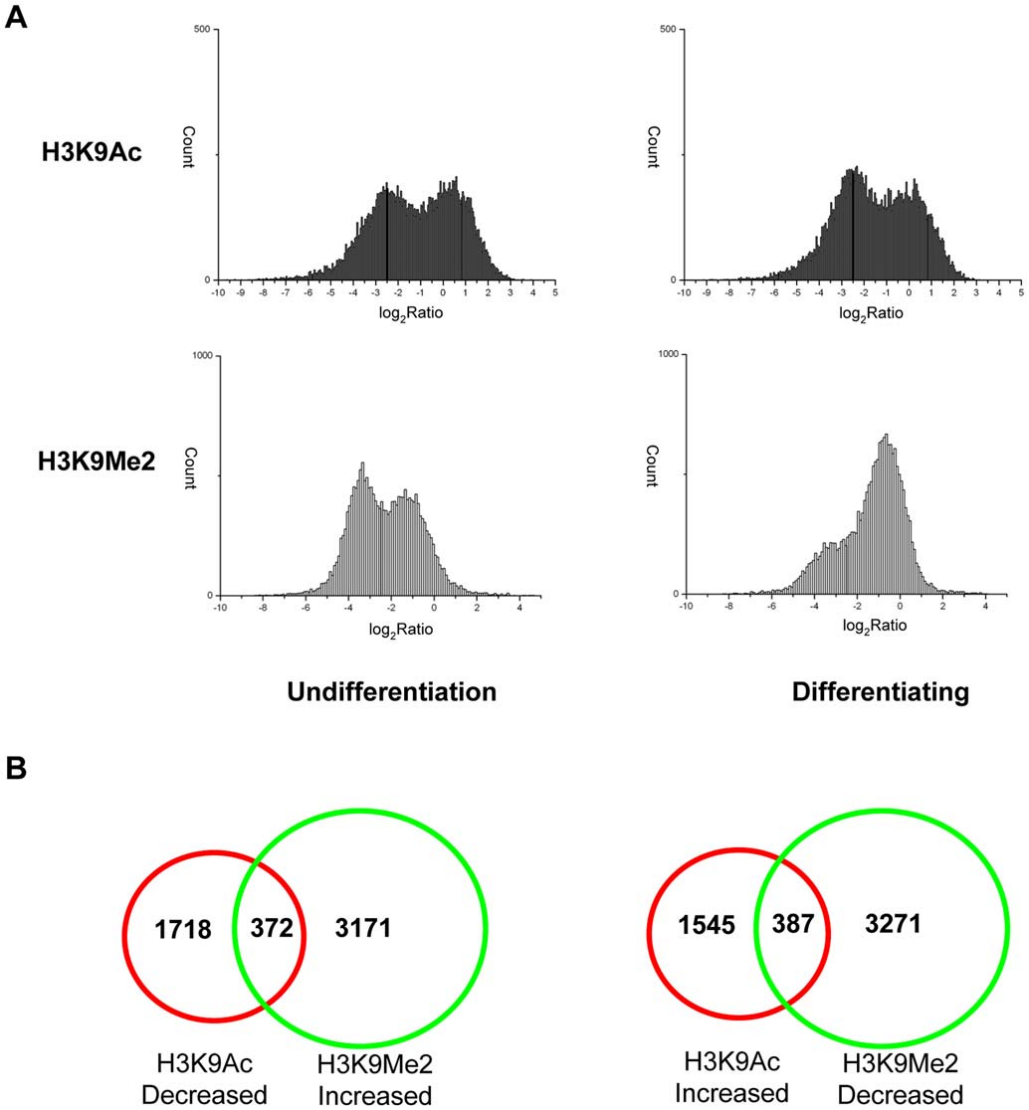
Genome-wide identification of the gene enriched with H3K9Ac and H3K9Me2 upon MSC osteogenic differentiation. (A) When the normalized mean IP/Input log ratios are plotted as density histograms, two populations of loci are apparent: the left peak represents the non-enriched DNA fragments and the peak at the right side corresponds to the enriched DNA fragments. (B) Venn diagrams of differential H3K9Ac versus H3K9Me2 changed genes.

Since the ChIP-on-chip results revealed that both H3K9Ac and H3K9Me2 were changed globally at day 7 of osteogenic induction, we wanted to further examine the genome-wide distributions of H3K9Ac and H3K9Me2 at gene promoter regions upon MSC osteogenic differentiation. In this study, the ChIP-DSL H20K promoter array (Aviva) was employed to generate H3K9Ac and H3K9Me2 profiles of gene promoter regions before and after MSC osteogenic differentiation. For the promoter array, each probe corresponded to a proximal promoter region from +200 bp to −800 bp relative to the transcription start sites, which contains>95% of known binding sites for transcription factors in human. Before hybridization of the H20K promoter arrays, control experiments were carried out to confirm the specificity and efficiency of the antibody and ChIP assay (Figure S1). MSCs were induced for osteogenesis for 7 days as previously described [10], and ChIP-on-chip assays were performed with antibodies against H3K9Ac and H3K9Me2 by co-hybridizing differentially labeled ChIP-enriched and total input DNAs to human promoter arrays. When we create an IP/Input log ratio density plot, the tail on the right distribution of log IP/Input was asymmetric with a heavy tail corresponding to enriched group [30]. The ChIP-on-chip data revealed that the gene promoters enriched with H3K9Ac was decreased slightly (Figure 3A), whereas the number of promoters enriched with H3K9Me2 was increased evidently after osteogenic induction (Figure 3A).

Moreover, by comparing the H3K9Ac and H3K9Me2 profiles at gene promoter regions between day 0 and day 7 cells, 1932 and 3543 gene promoters were found to exhibit more than 2-fold increased enrichments in H3K9Ac and H3K9Me2, respectively (Figure 3B and Table S2). Meanwhile, the promoter regions of 2090 genes and 3658 genes were decreased more than 2 folds in H3K9Ac and H3K9Me2 enrichments, respectively (Figure 3B and Table S2).

### Position weight matrix analysis of the promoter motifs of genes regulated by H3K9 modifications upon osteogenic differentiation

To characterize the gene promoters that displayed differential enrichments of H3K9Ac and H3K9Me2 upon the differentiation process, the position weight matrix (PWM) analysis of the transcription factor binding sites was carried out. The promoter sequences (from +200 bp to −800 bp relative to the transcription start sites) of these genes (Table S2) were uploaded to the *Cis*-regulatory Element Annotation System (CEAS, http://ceas.cbi.pku.edu.cn/ *)*. As the results, the motifs with top 10 quantity hits at gene promoter regions were listed (Table 1, Table 2). Specifically, it can be seen from the motif lists that a large number of vitamin D receptor potential binding sites (VDREs) (Table 1, Table 2) were detected at promoters of genes in H3K9Ac-decreased and H3K9Me2-increased groups; however, few VDREs were found in H3K9Ac-increased and H3K9Me2-decreased groups (Table 1, Table 2). Therefore, we proposed that VDR may possibly play as a regulator of deacetylation and dimethylation of H3K9 in MSC differentiation.

**Table 1:** Top 10 motifs of promoters underwent changes in H3K9Ac.

H3K9Ac Increased			H3K9Ac Decreased		
Enriched Motif	Hits	p-value	Enriched Motif	Hits	p-value
M00986.Churchill	3602	4.46E-245	M00448.Zic1	3268	2.23E-190
M00428.E2F-1	3129	2.05E-255	M00450.Zic3	3122	1.10E-134
M00716.ZF5	2807	0.00E+00	M00378.Pax-4	3120	2.06E-144
AP2alpha	2308	6.24E-187	AP2alpha	2958	0
M00803.E2F	2056	0	M00449.Zic2	2904	1.34E-115
M00333.ZF5	1818	3.14E-108	M00033.p300	2831	3.48E-92
M00470.AP-2gamma	1382	2.96E-119	M00961.VDR	2741	6.05E-97
M00431.E2F-1	1216	4.28E-112	M00098.Pax-2	2637	3.81E-134
M00008.Sp1	1192	4.68E-94	M00497.STAT3	2632	1.40E-102
Elk-1	1055	3.32067E-44	M00333.ZF5	2366	8.12E-304

**Table 2:** Top 10 motifs of promoters underwent changes in H3K9Me2.

H3K9Me2 Increased			H3K9Me2 Decreased		
Enriched Motif	Hits	p-value	Enriched Motif	Hits	p-value
M00378.Pax-4	5675	4.44E-264	M00448.Zic1	5805	3.38E-276
M00450.Zic3	5564	7.29E-223	M00450.Zic3	5767	1.61E-229
M00448.Zic1	5536	6.25E-254	M00378.Pax-4	5702	2.24E-234
M00961.VDR	4947	7.02E-170	AP2alpha	4969	0
AP2alpha	4879	0	M00497.STAT3	4749	5.43E-153
M00497.STAT3	4861	4.60E-202	M00098.Pax-2	4588	6.96E-172
M00098.Pax-2	4592	2.86E-200	M00008.Sp1	2439	1.25E-261
M00008.Sp1	2376	2.84E-261	M00723.GAGA	2170	1.37E-88
M00723.GAGA	2183	7.30E-105	Elk-1	2067	1.13E-115
Elk-1	2159	1.55E-154	M00446.Spz1	2043	5.86E-152

Enrichments of H3K9Ac and H3K9Me2 matrices in the promoter sequences (−800 to +200 relative to transcription start site) of target genes were analyzed by using the cis-regulatory element annotation system (CEAS).

**Figure 4:**
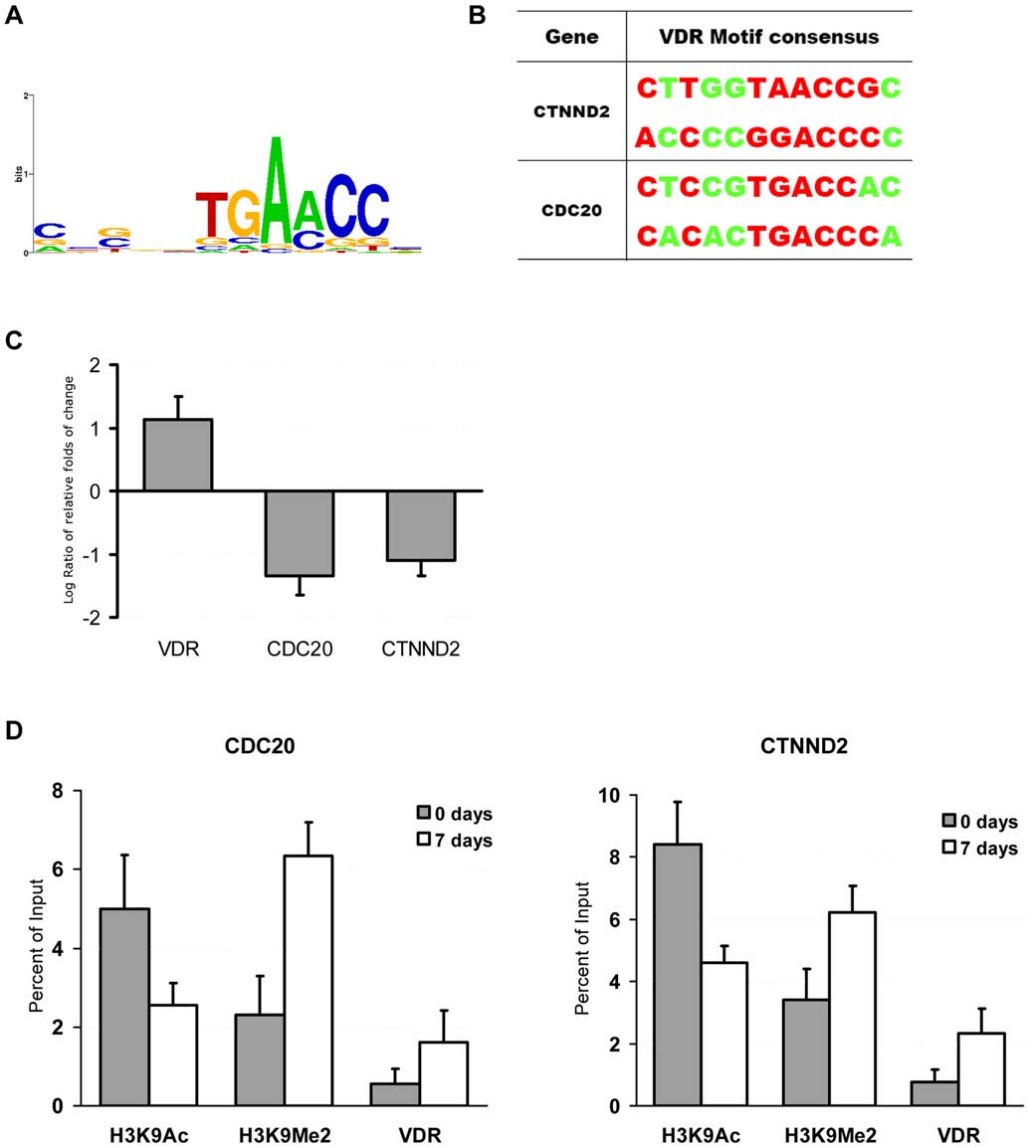
Position weight analysis of the promoters of genes regulated by H3K9 modifcations upon osteogenic differentiation. (A) A computational screening for the enriched motifs of vitamin D receptor binding regions (VDRE) with nucleotide bias shown using Weblogo (http://weblogo.berkeley.edu/). (B) The potential VDR binding elements at the promoter regions of CDC20 and CTNND2. (C) Real-time RT-PCR data showing that VDR was up-regulated, whereas CDC20 and CTNND2 were down-regulated 7 days after osteogenic induction. (D) Real-time ChIP-PCR indicating that the enrichments of VDR and H3K9Me2 were increased in CDC20 and CTNND2 promoter regions, whereas H3K9Ac was decreased upon MSC osteoblastic differentiation. Standard error bars of three individual experiments are indicated.

To validate our hypothesis, CDC20 and CTNND2 (catenin-δ) were selected for real-time RT-PCR and ChIP assay validation, which resulted in discovery of several potential VDREs in gene promoter regions (Figure 4A and B). Moreover, both CDC20 and CTNND2 exhibited more than 2 folds down-regulation upon the differentiation process, as shown by the microarrays (Table S3). Real-time RT-PCR demonstrated that VDR was up-regulated, whereas CDC20 and CTNND2 were down-regulated in MSC differentiation (Figure 4C). Also, real-time ChIP-PCR revealed that the enrichment of VDR was increased at the promoter regions of CDC20 and CTNND2 (Figure 4D). Overall, these data have led us to speculate that VDR played a trans-repression role in MSC osteogenic differentiation.

### Comparison of gene expression patterns with promoter H3K9 modification profiles in MSC osteogenic differentiation

**Figure 5:**
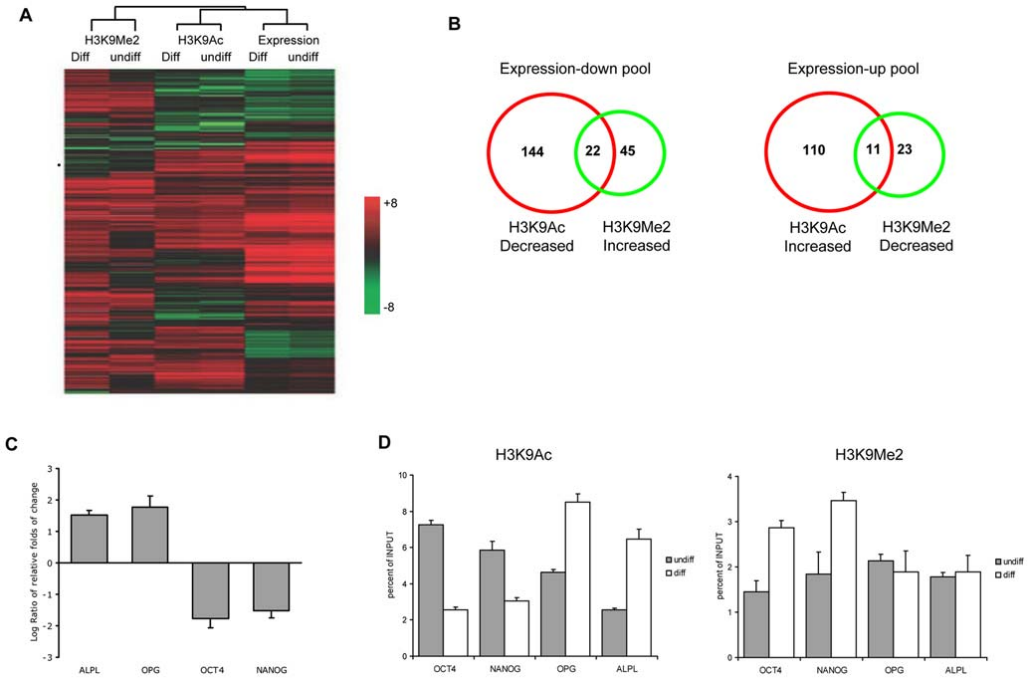
Comparative analyses of the data from gene expression patterns and from promoter H3K9 modification profiles. (A) Hierarchical cluster analysis of the enrichments of H3K9Ac/H3K9Me2, and the data of gene expression upon MSC osteogenic differentiation. (B) Venn diagrams of H3K9Ac-decreased genes versus H3K9Me2-increased genes in expression-down pool, and H3K9Ac- increased genes versus H3K9Me2-decreased genes in expression-up pool. (C) Real-time RT-PCR verification of gene expression of OCT4, NANOG, OPG and ALPL. The relative expression values were normalized against GAPDH. (D) Real-time ChIP-PCR verification of H3K9Ac and H3K9Me2 of promoter regions of OCT4, NANOG, OPG and ALPL upon MSC osteogenic differentiation. Standard error bars of three individual experiments are indicated.

It has been known that changes in H3K9Ac and H3K9Me2 modifications can influence the chromatin structure and affect gene expression [31], and these epigenetic modifications in specific loci could be critical for determining the fate of stem cells [20]. To determine the effects of changes of H3K9Ac and H3K9Me2 at gene promoter regions upon human MSC osteogenic differentiation, we performed a gene expression microarray analysis. From the expression microarray, we found that 1041 genes were up-regulated and 866 genes were down-regulated (Table S3). And then, we analyzed data from the ChIP-on-chip and that from expression microarrays in combination, which involved approximate 14000 known genes. To determine the relationship between H3K9Ac/H3K9Me2 and gene expression, hierarchical cluster analysis was performed. The results revealed that approximately 60% of the expressed genes were enriched with H3K9Ac in their promoter regions, while more than 70% of the under-expressed genes were enriched with H3K9Me2 (Figure 5A). The Venn diagram showed that 166 genes were down-regulated by deacetylation in H3K9 and 121 genes were up-regulated by hyperacetylation in H3K9; and meanwhile 67 genes were down-regulated by increased dimethylation and 34 genes were up-regulated by decreased dimethylation in H3K9 upon MSC osteogenic differentiation (Figure 5B).

To validate the results above, real-time RT-PCR and ChIP-PCR were carried out for randomly selected genes that were regulated by changes of H3K9Ac and H3K9Me2 at promoter regions shown in microarrays. The results indicated that the real-time PCR data correlated very well with that shown in the promoter and expression microarrays (Figure S2 and Table S4). Among the genes tested, we found that the stemness genes OCT4 and NANOG were down-regulated by deacetylation and dimethylation of H3K9 at promoter regions (Figure 5C and D); whereas the osteogenic specific genes OPG and ALPL were up-regulated with acetylated and de-dimethylated H3K9 at their promoters (Figure 5C and D).

### Pathway ontology analysis

**Figure 6:**
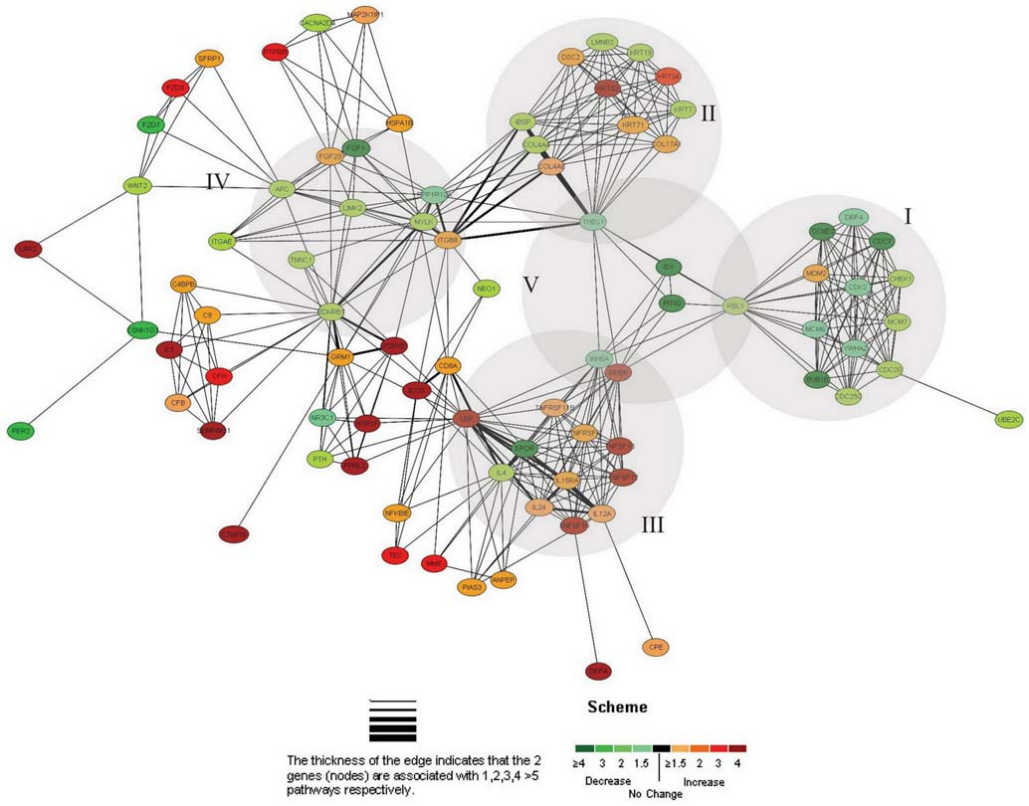
An informational network of the genes regulated by H3K9Ac and H3K9Me2 upon MSC osteogenic differentiation. The informational pathway networks were generated by using BioRag (*http//:* www.biorag.org). The most affected pathways are the cell cycle pathway (cluster I), the cell communication (cluster II), the cytokine-cytokine receptor interaction (cluster III), the regulation of actin cytoskeleton (cluster IV), and the TGF-beta signaling pathway (cluster V)

To further clarify the functional ontology and networks related to dynamic changes of enrichments of H3K9Ac and H3K9Me2 upon MSC osteogenic differentiation, we searched for the pathway association and connectivity between putative target genes, by using the software of pathway miner of the “Bio-resource for Array Gene System” (Biorag, http://www.biorag.org/). This software exploits a highly comprehensive database established from the literature to determine direct and indirect interactions between genes of interest, thereby identifying cellular pathways associated with a particular set of genes. GenBank accessions of these genes (Table S4) were uploaded to pathway miner of Biorag, and the gene lists and association network based on cellular and regulatory pathway data were generated (Figure 6). The analysis showed that differentially expressed genes regulated by changes of H3K9Ac and/or H3K9Me2 were involved in a number of cellular pathways as revealed from Kegg pathway database (Table S5). Specifically, we found that genes down-regulated by changes of H3K9Ac and H3K9Me2 at promoter regions upon MSC osteogenic differentiation principally belonged to cell cycle pathway, regulation of actin cytoskeleton pathway, and TGF-beta signaling pathway, etc. (Table S5). In contrast, genes up-regulated by changes of H3K9Ac and H3K9Me2 were primary distributed to cytokine-cytokine receptor interaction, complement and coagulation cascades and Jak-STAT signaling pathways (Table S5). Additionally, a network was generated depicting the functional relationships implicated in these pathways. Figure 6 illustrates such a network that centers largely on such genes as THBS1, PITX2, ID1, RBL1 and INHBA involved in TGF-beta signaling pathways, linking cell cycle, cell communication and cytokine-cytokine receptor interaction pathways together.

## Discussion

Recently, studies of the adult stem cells provide ample evidence that chromatin remodeling functions in universal differentiation events [32]. A prevailed view of development biology holds that the commitment of primitive multi-potent cells to specific lineages is mediated by key transcription factors that activate downstream tissue-specific genes [32]. Nevertheless, latest evidence shows that heterochromatin formation can also be an important mechanism for lineage restriction during development. For instance, the tri-methylation level of histone H3, lysine 9 (H3K9Me3) was shown to be increased dramatically during C2C12 cell line myogenesis, which subsequently led to a large-scale heterochromatin reorganization [29], [33]. And the researchers discovered that following differentiation from the activated B cell stage, chromatin in plasma cells became condensed as DAPI-dense facultative heterochromatin [34]. It seems likely that formation of facultative heterochromatin during cell differentiation involves some of the silencing mechanisms that affect gene expression. In this study, western blot and immunofluorescence data demonstrated that H3K9Ac was decreased and H3K9Me2 was increased globally upon MSC osteogenic differentiation (Figure 2A and B). Moreover, ChIP-on-chip data revealed that the number of genes with high acetylated H3K9 promoters was decreased, whereas the number of genes with high dimethylated H3K9 promoters was increased significantly 7 days after osteogenic induction (Figure 3). There have been indications that hypoacetylation of H3K9 and high dimethylated H3K9 were associated with facultative heterochromatin assembly resulting in transcriptional suppression [19]. Thus, our results imply that deacetylation and dimethylation of H3K9 may cause the formation of facultative heterochromatin at specific gene promoter regions, which may be significant to MSC osteogenic differentiation through some silencing mechanisms.

A noticeable point arising from this study has been that, by investigating the characteristics of promoter sequences of genes that underwent H3K9Ac or H3K9Me2 changes during MSC osteogenic differentiation, large numbers of vitamin D receptor elements (VDRE) were found at gene promoter regions in both H3K9Ac-decreased and H3K9Me2-increased groups (Table 1, Table 2), whereas few VDREs were found in H3K9Ac-increased and H3K9Me2-decreased groups (Table 1, Table 2). These results suggested that VDR may be a potential regulator for mediating deacetylation and dimethylation of H3K9 at specific gene promoter regions in MSC osteogenic differentiation. VDR is known as a member of the nuclear receptor (NR) gene superfamily, and acts as a ligand-inducible transcriptional factor [35]. It has been known that VDR could induce osteogenic differentiation through up-regulating bone-specific genes [36]. Nevertheless, several recent observations revealed that VDR could also repress transcription of genes in a ligand-dependent manner through chromatin remodeling of gene promoter regions [37]–[39]. In this study, we found that VDR was up-regulated more than 2 folds, whereas CDC20 and CTNND2 were down-regulated (Figure 4C) with deacetylated and dimethylated H3K9 at promoter regions. Simultaneously, the enrichment of VDR at promoter regions of CDC20 and CTNND2 were increased evidently upon MSC osteogenic differentiation (Figure 4D). CDC20 is known as a cell cycle regulator, and CTNND2 is initially identified as a neural-specific protein [40], [41]. The suppression of genes mediated by VDR participated in cell cycle arrest and the selective silencing of other lineage-specific gene in MSC osteogenic differentiation. Based on these data, we propose that VDR may possibly play dual roles in both trans-activation and trans-repression in MSC osteogenic differentiation; however, the potential trans-repression mechanisms in details were still not clearly understood. Recently, a model of ligand-induced trans-repression function of VDR was put forward [38], [39], in which ATP-dependent chromatin remodeling complex WINAC was required for the ligand-bound vitamin D receptor (VDR)-mediated trans-repression of the 25(OH)D_3_ 1α-hydroxylase genes. Accordingly, we speculate that this mechanism may potentially be effective in our MSC differentiation system, and the detailed mechanisms warrant further investigations.

In this study, we have exploited a pathway functional genomics approach to gain entry into epigenetic-related network, and attempted to bridge the gaps between genes, histone modifications and MSC osteogenic differentiation. Our pathway ontology analysis revealed that the differentially expressed genes that were regulated by changes of H3K9Ac and/or H3K9Me2 modifications were involved in a number of cellular pathways. Specifically, we demonstrated that genes down-regulated by deacetylation and dimethylation of H3K9 were involved in cell cycle related pathway, actin cytoskeleton regulation pathway (Table S5), whereas genes up-regulated by changes of H3K9Ac and H3K9Me2 were primary distributed to cytokine-cytokine receptor interaction (Table S5). Several studies implicated that differentiation of stem cells required the withdrawal from the cell cycle and re-establishment of a program of gene expression leading to the elaboration of a specialized phenotype [42]–[44]. Also, it was reported that rapid disruption of the actin cytoskeleton altered the morphology of MSC upon the induction of neuronal differentiation [45]. Moreover, cytokines function as a positive control for switching stem cells from a self-renewal to a differentiation stage [46]. These data are in accordance with our results, suggesting that dynamic changes of H3K9 modifications participated in regulation of biological events essential to MSC osteogenic differentiation. In addition, we observed that the osteogenic specific genes, OPG and ALPL, were activated by alternations of H3K9Ac and H3K9Me2 at promoter regions, whereas the stemness genes, OCT4 and NANOG, were trans-repressed by changes of H3K9Ac and H3K9Me2 upon MSC osteogenic differentiation (Figure 5C and D).

Furthermore, our work has also established a relationship network of the pathways regulated by changes of H3K9Ac and H3K9Me2 (Figure 6). One advantage of assessing gene-gene interaction network is that the characterization of the pathways can be based not only on the functions of target genes but also on those interacting genes. From this network, we noticed that genes involved in TGF-beta signaling pathway, such as THBS1, PITX2, ID1, RBL1 and INHBA, were at the center of the network, linking cell cycle, cell communication and cytokine-cytokine receptor interaction pathways together (Figure 6). In addition, it has been reported that TGF-beta-1 could inhibit MSC osteogenic differentiation [7]. Therefore, we propose that the H3K9Ac- and H3K9Me2-mediated gene silencing in TGF-beta signaling pathway may play a crucial role in MSC osteogenic differentiation. Besides, it is noted that these pathways in the network were also connected with one another through particular genes (Figure 6). The information presented above has provided us with important clues for further investigations into the interactive pathways as well as the functional and biological relevance of the targets of acetylation and dimethylation of H3K9.

In summary, data included in this report have depicted a genome-wide blueprint of H3K9Ac/H3K9Me2 modifications and expression profiles for human MSC osteogenic differentiation. Additional studies should yield further insights into the dynamics and hierarchy of epigenetic regulation in MSC osteogenic differentiation.

## Materials and Methods

### Ethics Statement

To obtain primary cultures, human bone marrow MSCs were derived from iliac crest marrow aspirates of healthy donor. A written consent for use of the bone marrow MSCs was presented by the donor.

The permission to use human bone marrow mesenchymal stem cells in this study was granted by Peking University Health Science Center's Ethical Committee.

### Isolation, culture and osteogenic differentiation induction of human bone marrow mesenchymal stem cells

Ten mL bone marrow from the donor was diluted 1∶2 with PBS and loaded over 5 mL Histopaque (Sigma). Cells were harvested from the interface after centrifugation at 2000 rpm for 20 min and washed with PBS. Cells were re-suspended in Modified Eagle's Medium of Alpha (α-MEM, Gibco) containing 10% fetal bovine serum (Hyclone), 100 U/mL of penicillin, 100 μg/mL streptomycin and 2 mM L-glutamine, and plated in a flask at the density of 3×10^5^ cells/mL. Non-adherent cells were discarded after cultivation for 48 h. The adherent cells were washed twice and cultured for 10–14 days until cell clones were formed. Cells were analyzed by flow cytometry to confirm their identity of MSCs. Cells were then plated at a density of 1×10^5^ cells/cm^2^ on amniotic membrane and expanded until 90% confluence [47].

Osteogenic differentiation medium was prepared by supplementing the growth medium with 50 mg/mL ascorbic acid, 1.5 mg/mL β-glycerophosphate (Sigma), and 10^−8^ M dexamethasone (Sigma). Growth and differentiation media were replaced twice a week and ascorbic acid was added to the differentiation medium every other day [10].

### Alkaline phosphatase enzyme histochemistry and von kossa Staining

To detect alkaline phosphatase activity, cells were stained with 1 mg/mL Fast Red^TR^ (Sigma-Aldrich) and 0.2 mg/mL napthol AS-MX phosphate (Sigma-Aldrich), dissolved in 1 mL N, N-dimethylformamide (Sigma-Aldrich) in 0.1 M Tris buffer at pH 9.2, and then fixed in 4% paraformaldehyde.

Calcium deposition was identified by using the von kossa staining te**c**hnique by adding 1% silver nitrate at room temperature for 60 min under strong light, followed by 2.5% sodium thiosulphate for 5 min.

### Analysis of expression microarray and quantitative real-time RT-PCR

Total RNA was isolated from cells using the Trizol reagents (Invitrogen). The human genome 70-mer oligonucleotide microarrays were obtained from CapitalBio, and the hybridization was done by the CapitalBio Company service Corporation (Beijing, China). The microarrays were scanned on a GenePix Pro4.0 scanner (Axon Instrument). Statistical analyses were performed by using the statistical software R; all the scripts that were used are available on request, and a space- and intensity-dependent normalization based on a LOWESS program was employed [48]. For each tests and control samples, two hybridizations were performed by using a reverse fluorescence strategy, and 1.8-fold averaged over the two biological replicates was set for significant change between differentiation and undifferentiating conditions of MSCs. Additionally, a nominal *p* value threshold of<0.05 was used to select differentially expressed genes for further analyses. Briefly, as there is a reverse fluorescence strategy in expression analysis, we firstly averaged the two ratio replicates after LOWESS normalization, and then used a corrected method provided by Chen et al [49] to quantify the significance of observed differences in expression ratios. Different from Chen's, here we proposed a robust method to estimate the c value:
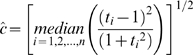
where 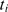 is the *i*th gene expression ratio. Using the estimation of c, the *p* value was calculated as follows: (T = R/G)
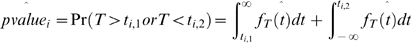
where 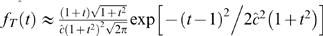, one of the 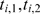
was calculated by the observation, the other one had the same density value and calculated through 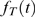.

Microarray data reported in the manuscript is deposited to Array-express, and the expression microarray accession number is A-MEXP-1567 (in accordance with MIAME guidelines).

Total RNA was reverse transcribed to cDNA. Three independent cDNA samples were analyzed by using real-time RT-PCR on an ABI PRISM7000 Sequence Detection System (Applied Biosystems), and the GAPDH gene was used as the internal reference for normalizing the variations in the quality and the amount of total cDNA. The primer sequences are shown in Table S1.

### Antibodies and ChIP assays

Anti-acetylated H3K9 (07-352) and anti-dimethylated H3K9 (07-212) antibodies were purchased from Upstate Biotechnology. Anti-VDR was obtained from Abcam (ab54387). Cells were crosslinked with formaldehyde and subjected to chromatin immunoprecipitation (ChIP) assay as previously described [50]. Briefly, 1×10^6^ cells were treated with 2% formaldehyde in IMDM medium for 10 min at 37°C to crosslink protein-DNA. To stop the reaction, glycine (125 mM final) was added. After washing 4 times with cold PBS, cells were lysed by incubating in nucleus lysis buffer. The lysate was sonicated with a UibraCell TM-130 sonicator. After centrifugation at 12000 rpm for 10 min, the supernatant was diluted 1∶10 in ChIP dilution buffer. The chromatin solution was precleared with 50 μL of protein A-agarose beads (Upstate Biotechnology). The soluble fraction was collected and 5 μg of anti-acetyl-histone H3K9 (Upstate Biotechnology), anti-dimethyl-histone H3K9 (Upstate Biotechnology), and anti-VDR (Sigma, F3165) antibodies were added. After immunoprecipitation, the precipitated complexes were collected by adding 60 μL of protein A-agarose beads. After washing, freshly prepared elution buffer (1%SDS, 0.1 M NaHCO_3_) were added, and the crosslinking was reversed at 65°C for 4 h. DNA was recovered by Proteinase K digestion, phenol/chloroform extraction, and ethanol precipitation.

Real-time quantitative PCR was performed on an ABI PRISM7000 Sequence Detection System (Applied Biosystems), and the total input was used as the internal reference for normalization. The primer sequences are shown in Table S1.

### ChIP-DSL and ChIP-chip assays

For ChIP-DSL assays, both input (5% of total DNA) and IP (antibody-enriched DNA) were randomly biotinylated by using a kit (Vector Laboratories) according to the manufacturer's instructions. All the T7-linked oligonucleotides were kinased and then mixed with all T3-linked oligonucleotides. For each reaction, we used 0.1 pmol per oligonucleotide in a pool suspended in 10 μL of TE buffer. The procedures for oligonucleotide annealing, solid phase selection, ligation and PCR amplification were as described [51], except *Taq* ligase was used in place of T4 ligase to improve ligation specificity. Input DNA was labeled with Alexa Fluor 647 and the immunoprecipitated DNA with Cy3. PCR products were mixed, denatured, and hybridized to the 40-mer Hu20K array. Slides were scanned on the GenePix Pro4.0 (Axon Instrument). The Hu20K array and the associated assay kit with detailed instruction are commercially available from Aviva Systems Biology [26], [27].

### ChIP-chip data analysis

Due to both experimental and technical variability, the signal must be standardized for proper comparison among experiments. This can be done using the stochastic part of log IP/Input distributions corresponding to the regions in which there is no binding [30]. The distribution of log IP/Input may be asymmetric with a heavy tail on the right corresponding to enrich [30]. The frequencies of loci with different IP signal intensities were modeled using EM algorithm to separate loci into groups with enriched and non-enriched [30]. The LOWESS program was used to measure the variability between different ChIP-on-chip arrays[52], [53]. For identification of H3K9 targets that are differentially acetylated and methylated between differentiation and undifferentiating conditions of MSCs, 2-fold was set for the significant change of H3K9Ac and H3K9Me2. Similarly, the nominal *p* value threshold of<0.05 was also used to distinguish the modification changed genes. Briefly, two channel image data ratio (Cy5/Cy3) were normalized by LOWESS method, and the *p* value calculation was implemented using the same method as in the expression analysis.

Microarray data reported in the manuscript is deposited to array-express, ChIP-chip accession number E-MEXP-2134 (in accordance with MIAME guidelines).

### Promoter motif analysis

Promoter sequences (from −800 to +200) of genes undergoing changes of H3K9Ac and H3K9Me2 modifications in MSC osteogenic differentiation were analyzed by using the *Cis*-regulatory Element Annotation System (CEAS, http://ceas.cbi.pku.edu.cn/) to search for enrichments of position weight matrices (PWMs) of transcription factor-binding motif in TRANSFAC [54].

### Pathway analysis

Gene ontology and pathway analysis were performed by using pathway miner of the Bio-resource for Array Gene System (Biorag, http://www.biorag.org/). GenBank accessions were uploaded to Biorag, and the gene lists and association networks based on cellular and regulatory pathway data were generated [55].

## Supporting Information

Figure S1 (A) Verification of specificity of antibodies. ChIP was performed with the antibodies specific for H3K9Ac and H3K9Me2 and immuno-detected by western blot with the antibody against H3 without reverse-linking. (B) Validation of efficiency of traditional ChIP assay. The antibody specific to H3K9Ac was used, and β-actin gene promoter region was set as a positive controal.
(0.30 MB TIF)

Figure S2 Validation of expressiomn microarray and ChIP-chip data by real time PCR. (A) Real time RT-PCR verification of gene regulated by changes of H3K9Ac and H3K9Me2 at promoter regions upon MSC osteogenic differentiation. The relative expression values were normalized against GAPDH. Real time ChIP-PCR verification of H3K9Me2 (B) and H3K9Ac (C) at promoter regions of the selected genes upon MSC osteogenic differentiation. Standard error bars of three individual experiments are indicated.
(0.49 MB TIF)

Table S1 primers for real time PCR
(0.02 MB XLS)

Table S2 the genes with changes of H3K9 modifications
(1.04 MB XLS)

Table S3 differentially expressed genes
(0.46 MB XLS)

Table S4 the genes regulated by changes of H3K9 modificaions
(0.17 MB XLS)

Table S5 Pathway ontology classification of differentially expressed genes regulated by H3K9Ac and H3K9Me2
(0.18 MB DOC)
