# Supplementary figures and images for: Genome-Wide Analysis of Histone H3 Lysine9 Modifications in Human Mesenchymal Stem Cell Osteogenic Differentiation
Source: PLoS One. 2009 Aug 27;4(8):e6792. doi: 10.1371/journal.pone.0006792 (PMC2729372; doi:10.1371/journal.pone.0006792)

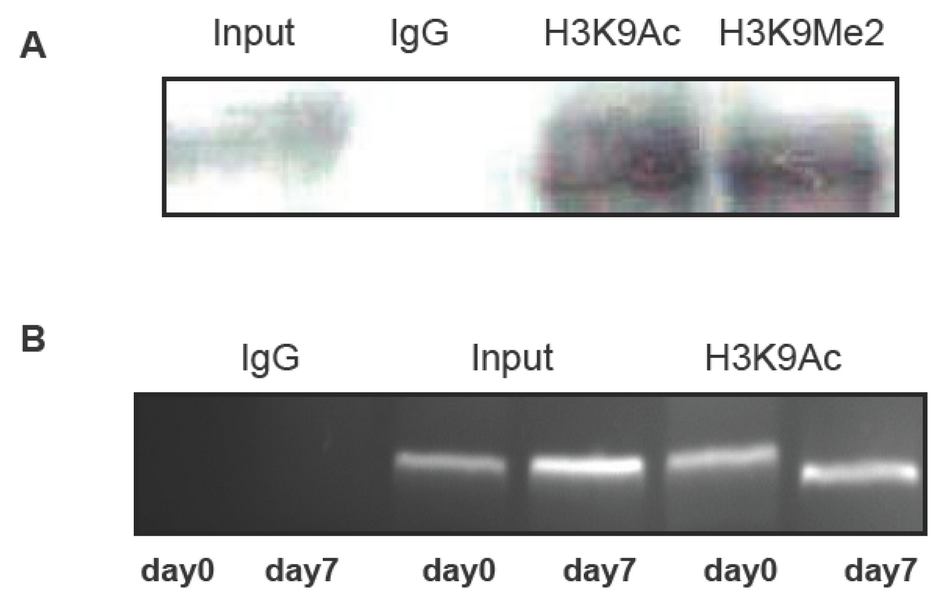

Supplement: Figure S1 — (A) Verification of specificity of antibodies. ChIP was performed with the antibodies specific for H3K9Ac and H3K9Me2 and immuno-detected by western blot with the antibody against H3 without reverse-linking. (B) Validation of efficiency of traditional ChIP assay. The antibody specific to H3K9Ac was used, and β-actin gene promoter region was set as a positive controal. (0.30 MB TIF) [file pone.0006792.s001.tif]

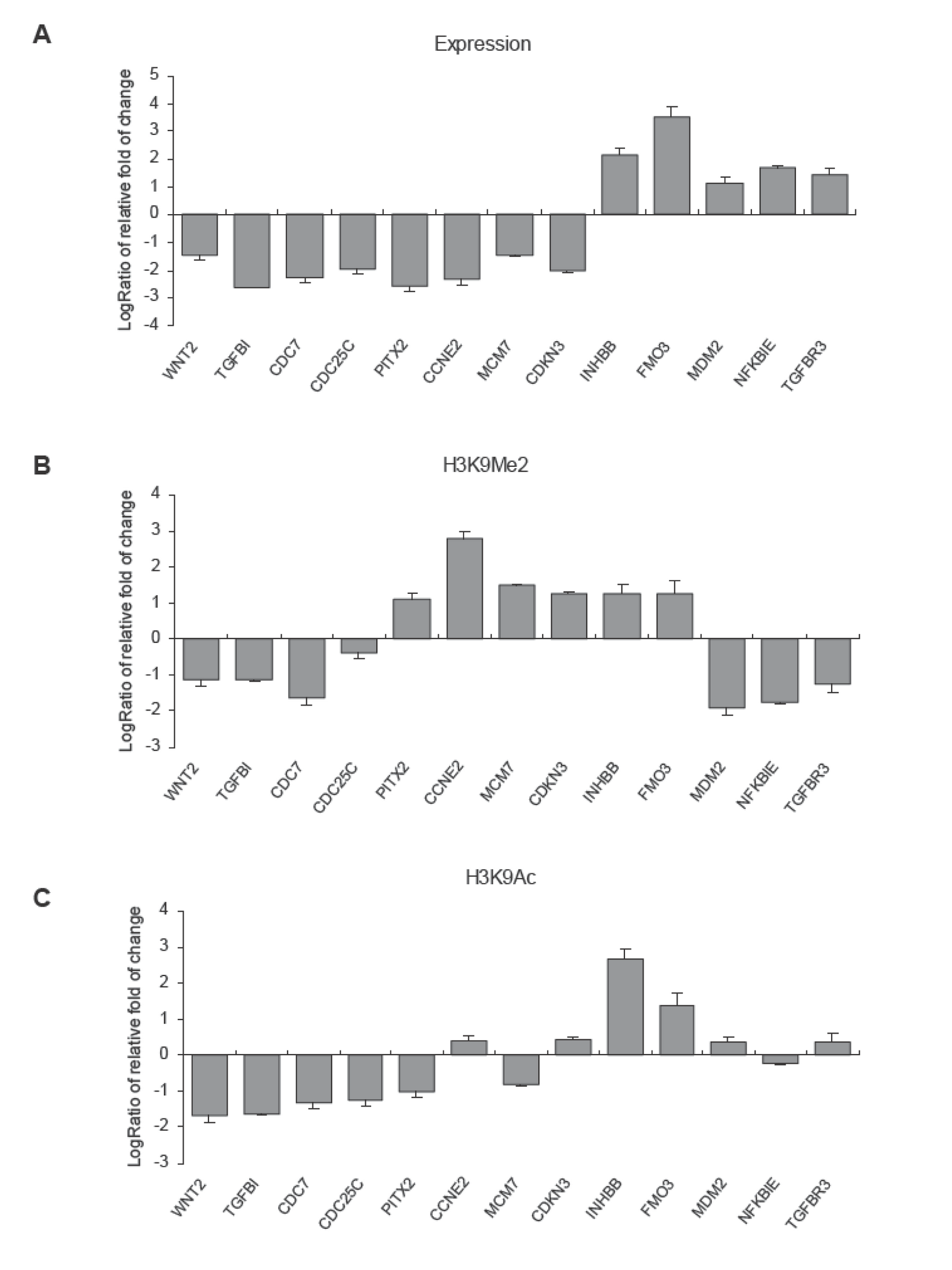

Supplement: Figure S2 — Validation of expressiomn microarray and ChIP-chip data by real time PCR. (A) Real time RT-PCR verification of gene regulated by changes of H3K9Ac and H3K9Me2 at promoter regions upon MSC osteogenic differentiation. The relative expression values were normalized against GAPDH. Real time ChIP-PCR verification of H3K9Me2 (B) and H3K9Ac (C) at promoter regions of the selected genes upon MSC osteogenic differentiation. Standard error bars of three individual experiments are indicated. (0.49 MB TIF) [file pone.0006792.s002.tif]
