# Supplementary material for: Genome-Wide Analysis of Histone H3 Lysine9 Modifications in Human Mesenchymal Stem Cell Osteogenic Differentiation
Source: PLoS One. 2009 Aug 27;4(8):e6792. doi: 10.1371/journal.pone.0006792 (PMC2729372; doi:10.1371/journal.pone.0006792)
Supplement: Table S5 — Pathway ontology classification of differentially expressed genes regulated by H3K9Ac and H3K9Me2 (0.18 MB DOC) [file pone.0006792.s007.doc]

| Expression Up-regulation Pool | | | | | Expression Down-regulation Pool | | | | |
| --- | --- | --- | --- | --- | --- | --- | --- | --- | --- |
| Pathway | Gene  Name | Gene  Accession | H3K9Ac up | H3K9Me2 down | Pathway | Gene  Name | Gene  Accession | H3K9Ac down | H3K9Me2 up |
| [Cytokine-cytokine](http://bach.biosci.arizona.edu/Pathway/?type=KEGG&file1=22726aaaaab&file2=22726aaaaac&logratio=Yes&mapno=hsa04060&pname=Cytokine-cytokine receptor interaction) | [TNFSF13](http://www.biorag.org/perl/biorag.pl?lid=8741) | NM_003808 | **+** |  | [Cell cycle](http://bach.biosci.arizona.edu/Pathway/?type=KEGG&file1=23099aaaaad&file2=23099aaaaae&logratio=Yes&mapno=hsa04110&pname=Cell cycle) | [CCNE2](http://www.biorag.org/perl/biorag.pl?lid=9134) | NM_057749 | **+** | **+** |
| receptor | [IL12A](http://www.biorag.org/perl/biorag.pl?lid=3592) | NM_000882 | **+** |  |  | [MCM6](http://www.biorag.org/perl/biorag.pl?lid=4175) | NM_005915 |  | **+** |
| interaction | [IL15RA](http://www.biorag.org/perl/biorag.pl?lid=3601) | NM_002189 | **+** | **+** |  | [RBL1](http://www.biorag.org/perl/biorag.pl?lid=5933) | NM_002895 |  | **+** |
|  | [TNFSF12](http://www.biorag.org/perl/biorag.pl?lid=8742) | NM_003808 | **+** |  |  | [CDC7](http://www.biorag.org/perl/biorag.pl?lid=8317) | NM_003503 | **+** |  |
|  | [INHBB](http://www.biorag.org/perl/biorag.pl?lid=3625) | NM_002193 | **+** |  |  | [DBF4](http://www.biorag.org/perl/biorag.pl?lid=10926) | NM_006716 |  | **+** |
|  | [IL24](http://www.biorag.org/perl/biorag.pl?lid=11009) | NM_006850 |  | **+** |  | [YWHAZ](http://www.biorag.org/perl/biorag.pl?lid=7534) | NM_003406 | **+** | **+** |
|  | [TNFRSF8](http://www.biorag.org/perl/biorag.pl?lid=943) | NM_001243 |  | **+** |  | [CHEK1](http://www.biorag.org/perl/biorag.pl?lid=1111) | NM_001274 |  | **+** |
|  | [TNFSF10](http://www.biorag.org/perl/biorag.pl?lid=8743) | NM_003810 | **+** | **+** |  | [BUB1B](http://www.biorag.org/perl/biorag.pl?lid=701) | NM_001211 |  | **+** |
|  | [LEP](http://www.biorag.org/perl/biorag.pl?lid=3952) | NM_000230 |  | **+** |  | [MCM7](http://www.biorag.org/perl/biorag.pl?lid=4176) | NM_005916 |  | **+** |
|  | [TNFRSF11B](http://www.biorag.org/perl/biorag.pl?lid=4982) | NM_002546 | **+** |  |  | [CDK2](http://www.biorag.org/perl/biorag.pl?lid=1017) | NM_001798 | **+** |  |
| [Cell](http://bach.biosci.arizona.edu/Pathway/?type=KEGG&file1=22726aaaaab&file2=22726aaaaac&logratio=Yes&mapno=hsa01430&pname=Cell Communication) | [COL17A1](http://www.biorag.org/perl/biorag.pl?lid=1308) | NM_000494 | **+** |  |  | [CDC25C](http://www.biorag.org/perl/biorag.pl?lid=995) | NM_001790 | **+** |  |
| Communication | [DSC2](http://www.biorag.org/perl/biorag.pl?lid=1824) | NM_004949 | **+** |  |  | CDC20 | NM_001255 | **+** | **+** |
|  | [KRT71](http://www.biorag.org/perl/biorag.pl?lid=112802) | NM_033448 |  | **+** | [Regulation of](http://bach.biosci.arizona.edu/Pathway/?type=KEGG&file1=23099aaaaad&file2=23099aaaaae&logratio=Yes&mapno=hsa04810&pname=Regulation of actin cytoskeleton) | [FGF1](http://www.biorag.org/perl/biorag.pl?lid=2246) | NM_000800 |  | **+** |
|  | [COL4A6](http://www.biorag.org/perl/biorag.pl?lid=1288) | NM_033641 |  | **+** | actin | [ITGAE](http://www.biorag.org/perl/biorag.pl?lid=3682) | NM_002208 | **+** |  |
|  | [KRT34](http://www.biorag.org/perl/biorag.pl?lid=3885) | NM_021013 |  | **+** | cytoskeleton | [PPP1R12A](http://www.biorag.org/perl/biorag.pl?lid=4659) | NM_002480 |  | **+** |
|  | [KRT32](http://www.biorag.org/perl/biorag.pl?lid=3882) | NM_002278 |  | **+** |  | [MYLK](http://www.biorag.org/perl/biorag.pl?lid=4638) | NM_053025 | **+** | **+** |
| [Complement and](http://bach.biosci.arizona.edu/Pathway/?type=KEGG&file1=22726aaaaab&file2=22726aaaaac&logratio=Yes&mapno=hsa04610&pname=Complement and coagulation cascades) | [CFH](http://www.biorag.org/perl/biorag.pl?lid=3075) | NM_000186 |  | **+** |  | [BDKRB1](http://www.biorag.org/perl/biorag.pl?lid=623) | NM_000710 |  | **+** |
| coagulation | [SERPING1](http://www.biorag.org/perl/biorag.pl?lid=710) | NM_000062 | **+** |  |  | [LIMK2](http://www.biorag.org/perl/biorag.pl?lid=3985) | NM_016733 | **+** | **+** |
| cascades | [CFB](http://www.biorag.org/perl/biorag.pl?lid=629) | NM_001710 | **+** |  | [Cell](http://bach.biosci.arizona.edu/Pathway/?type=KEGG&file1=23099aaaaad&file2=23099aaaaae&logratio=Yes&mapno=hsa01430&pname=Cell Communication) | [LMNB2](http://www.biorag.org/perl/biorag.pl?lid=84823) | NM_032737 | **+** | **+** |
|  | [C7](http://www.biorag.org/perl/biorag.pl?lid=730) | NM_000587 | **+** |  | Communication | [KRT7](http://www.biorag.org/perl/biorag.pl?lid=3855) | NM_005556 | **+** | **+** |
|  | [C4BPB](http://www.biorag.org/perl/biorag.pl?lid=725) | NM_000716 | **+** | **+** |  | [COL4A4](http://www.biorag.org/perl/biorag.pl?lid=1286) | NM_000092 | **+** |  |
|  | [C9](http://www.biorag.org/perl/biorag.pl?lid=735) | NM_001737 |  | **+** |  | [KRT19](http://www.biorag.org/perl/biorag.pl?lid=3880) | NM_002276 |  | **+** |
| [Neuroactive](http://bach.biosci.arizona.edu/Pathway/?type=KEGG&file1=22726aaaaab&file2=22726aaaaac&logratio=Yes&mapno=hsa04080&pname=Neuroactive ligand-receptor interaction) | [GRM1](http://www.biorag.org/perl/biorag.pl?lid=2911) | NM_000838 | **+** |  |  | [IBSP](http://www.biorag.org/perl/biorag.pl?lid=3381) | NM_004967 |  | **+** |
| ligand-receptor | [LEP](http://www.biorag.org/perl/biorag.pl?lid=3952) | NM_000230 |  | **+** |  | [THBS1](http://www.biorag.org/perl/biorag.pl?lid=7057) | NM_003246 | **+** | **+** |
| interaction | [FPRL2](http://www.biorag.org/perl/biorag.pl?lid=2359) | NM_002030 | **+** |  | [TGF-beta](http://bach.biosci.arizona.edu/Pathway/?type=KEGG&file1=23099aaaaad&file2=23099aaaaae&logratio=Yes&mapno=hsa04350&pname=TGF-beta signaling pathway) | [PITX2](http://www.biorag.org/perl/biorag.pl?lid=5308) | NM_000325 | **+** | **+** |
|  | [HTR1F](http://www.biorag.org/perl/biorag.pl?lid=3355) | NM_000866 | **+** | **+** | signaling | [ID1](http://www.biorag.org/perl/biorag.pl?lid=3397) | NM_002165 | **+** | **+** |
|  | [P2RX5](http://www.biorag.org/perl/biorag.pl?lid=5026) | NM_002561 |  | **+** | pathway | [RBL1](http://www.biorag.org/perl/biorag.pl?lid=5933) | NM_002895 |  | **+** |
| [Jak-STAT](http://bach.biosci.arizona.edu/Pathway/?type=KEGG&file1=22726aaaaab&file2=22726aaaaac&logratio=Yes&mapno=hsa04630&pname=Jak-STAT signaling pathway) | [PIAS3](http://www.biorag.org/perl/biorag.pl?lid=10401) | AK057853 | **+** |  |  | [INHBA](http://www.biorag.org/perl/biorag.pl?lid=3624) | NM_002192 | **+** |  |
| signaling | [IL12A](http://www.biorag.org/perl/biorag.pl?lid=3592) | NM_000882 | **+** | **+** |  | [THBS1](http://www.biorag.org/perl/biorag.pl?lid=7057) | NM_003246 | **+** | **+** |
| pathway | [IL15RA](http://www.biorag.org/perl/biorag.pl?lid=3601) | NM_002189 | **+** | **+** | [Focal adhesion](http://bach.biosci.arizona.edu/Pathway/?type=KEGG&file1=23099aaaaad&file2=23099aaaaae&logratio=Yes&mapno=hsa04510&pname=Focal adhesion) | [PPP1R12A](http://www.biorag.org/perl/biorag.pl?lid=4659) | NM_002480 |  | **+** |
|  | [IL24](http://www.biorag.org/perl/biorag.pl?lid=11009) | NM_006850 |  | **+** |  | [COL4A4](http://www.biorag.org/perl/biorag.pl?lid=1286) | NM_000092 | **+** |  |
|  | [LEP](http://www.biorag.org/perl/biorag.pl?lid=3952) | NM_000230 |  | **+** |  | [MYLK](http://www.biorag.org/perl/biorag.pl?lid=4638) | NM_053025 | **+** | **+** |
| [MAPK signaling](http://bach.biosci.arizona.edu/Pathway/?type=KEGG&file1=22726aaaaab&file2=22726aaaaac&logratio=Yes&mapno=hsa04010&pname=MAPK signaling pathway) | [PTPRR](http://www.biorag.org/perl/biorag.pl?lid=5801) | NM_002849 |  | **+** |  | [IBSP](http://www.biorag.org/perl/biorag.pl?lid=3381) | NM_004967 |  | **+** |
| pathway | [MAP2K1IP1](http://www.biorag.org/perl/biorag.pl?lid=8649) | NM_021970 | **+** |  |  | [THBS1](http://www.biorag.org/perl/biorag.pl?lid=7057) | NM_003246 | **+** | **+** |
|  | [FGF23](http://www.biorag.org/perl/biorag.pl?lid=8074) | NM_020638 |  | **+** | [Wnt signaling](http://bach.biosci.arizona.edu/Pathway/?type=KEGG&file1=23099aaaaad&file2=23099aaaaae&logratio=Yes&mapno=hsa04310&pname=Wnt signaling pathway) | [WNT2](http://www.biorag.org/perl/biorag.pl?lid=7472) | NM_003391 | **+** |  |
|  | [HSPA1B](http://www.biorag.org/perl/biorag.pl?lid=3304) | NM_005346 |  | **+** | pathway | [APC](http://www.biorag.org/perl/biorag.pl?lid=324) | NM_000038 | **+** |  |
| [T cell receptor](http://bach.biosci.arizona.edu/Pathway/?type=KEGG&file1=22726aaaaab&file2=22726aaaaac&logratio=Yes&mapno=hsa04660&pname=T cell receptor signaling pathway) | [CD8A](http://www.biorag.org/perl/biorag.pl?lid=925) | NM_001768 |  | **+** |  | [FZD7](http://www.biorag.org/perl/biorag.pl?lid=8324) | NM_003507 |  | **+** |
| signaling | [NFKBIE](http://www.biorag.org/perl/biorag.pl?lid=4794) | NM_004556 |  | **+** | [cytokine-cytokine](http://bach.biosci.arizona.edu/Pathway/?type=KEGG&file1=23099aaaaad&file2=23099aaaaae&logratio=Yes&mapno=hsa04060&pname=Cytokine-cytokine receptor interaction) | [EPOR](http://www.biorag.org/perl/biorag.pl?lid=2057) | NM_000121 | **+** |  |
| pathway | [TEC](http://www.biorag.org/perl/biorag.pl?lid=7006) | NM_003215 |  | **+** | receptor | [IL4](http://www.biorag.org/perl/biorag.pl?lid=3565) | NM_000589 | **+** |  |
|  | [ICOS](http://www.biorag.org/perl/biorag.pl?lid=29851) | NM_012092 |  | **+** | interaction | [INHBA](http://www.biorag.org/perl/biorag.pl?lid=3624) | NM_002192 | **+** |  |
| [Cell adhesion](http://bach.biosci.arizona.edu/Pathway/?type=KEGG&file1=22726aaaaab&file2=22726aaaaac&logratio=Yes&mapno=hsa04514&pname=Cell adhesion molecules (CAMs)) | [CD8A](http://www.biorag.org/perl/biorag.pl?lid=925) | NM_001768 |  | **+** | [Neuroactive](http://bach.biosci.arizona.edu/Pathway/?type=KEGG&file1=23099aaaaad&file2=23099aaaaae&logratio=Yes&mapno=hsa04080&pname=Neuroactive ligand-receptor interaction) | [NR3C1](http://www.biorag.org/perl/biorag.pl?lid=2908) | NM_000176 |  | **+** |
| molecules | [ITGB8](http://www.biorag.org/perl/biorag.pl?lid=3696) | NM_002214 |  | **+** | ligand-receptor | [BDKRB1](http://www.biorag.org/perl/biorag.pl?lid=623) | NM_000710 | **+** |  |
| (CAMs) | [ICOS](http://www.biorag.org/perl/biorag.pl?lid=29851) | NM_012092 |  | **+** | interaction | [PTH](http://www.biorag.org/perl/biorag.pl?lid=5741) | NM_000315 |  | **+** |
| [Hematopoietic](http://bach.biosci.arizona.edu/Pathway/?type=KEGG&file1=22726aaaaab&file2=22726aaaaac&logratio=Yes&mapno=hsa04640&pname=Hematopoietic cell lineage) | [CD8A](http://www.biorag.org/perl/biorag.pl?lid=925) | NM_001768 |  | **+** | [Calcium pathway](http://bach.biosci.arizona.edu/Pathway/?type=KEGG&file1=23099aaaaad&file2=23099aaaaae&logratio=Yes&mapno=hsa04020&pname=Calcium signaling pathway) | [TNNC1](http://www.biorag.org/perl/biorag.pl?lid=7134) | NM_003280 | **+** |  |
| cell lineage | [MME](http://www.biorag.org/perl/biorag.pl?lid=4311) | NM_007289 | **+** |  | signaling | [BDKRB1](http://www.biorag.org/perl/biorag.pl?lid=623) | NM_000710 | **+** |  |
|  | [ANPEP](http://www.biorag.org/perl/biorag.pl?lid=290) | NM_001150 |  | **+** |  | [MYLK](http://www.biorag.org/perl/biorag.pl?lid=4638) | NM_053025 | **+** | **+** |
| [Regulation of actin](http://bach.biosci.arizona.edu/Pathway/?type=KEGG&file1=22726aaaaab&file2=22726aaaaac&logratio=Yes&mapno=hsa04810&pname=Regulation of actin cytoskeleton) | [ITGB8](http://www.biorag.org/perl/biorag.pl?lid=3696) | NM_002214 |  | **+** | [ECM-receptor](http://bach.biosci.arizona.edu/Pathway/?type=KEGG&file1=23099aaaaad&file2=23099aaaaae&logratio=Yes&mapno=hsa04512&pname=ECM-receptor interaction) | [THBS1](http://www.biorag.org/perl/biorag.pl?lid=7057) | NM_003246 | **+** | **+** |
| cytoskeleton | [FGF23](http://www.biorag.org/perl/biorag.pl?lid=8074) | NM_020638 |  | **+** | interaction | [COL4A4](http://www.biorag.org/perl/biorag.pl?lid=1286) | NM_000092 | **+** |  |
| [Wnt signaling](http://bach.biosci.arizona.edu/Pathway/?type=KEGG&file1=22726aaaaab&file2=22726aaaaac&logratio=Yes&mapno=hsa04310&pname=Wnt signaling pathway) | [FZD3](http://www.biorag.org/perl/biorag.pl?lid=7976) | NM_017412 |  | **+** |  | [IBSP](http://www.biorag.org/perl/biorag.pl?lid=3381) | NM_004967 |  | **+** |
| pathway | [SFRP1](http://www.biorag.org/perl/biorag.pl?lid=6422) | NM_003012 | **+** |  |  |  |  |  |  |

^*^ “**+**” means genes differentially expressed regulated by change of H3K9Ac or H3K9Me2 upon MSC osteogenic differentiation.
